# Integrating shortest dependency path and sentence sequence into a deep learning framework for relation extraction in clinical text

**DOI:** 10.1186/s12911-019-0736-9

**Published:** 2019-01-31

**Authors:** Zhiheng Li, Zhihao Yang, Chen Shen, Jun Xu, Yaoyun Zhang, Hua Xu

**Affiliations:** 1 School of Computer Science and Technology, Dalian University of Technology, Dalian, 116024 China; 2 School of Biomedical Informatics, The University of Texas Health Science Center at Houston, Houston, TX 77030 USA

**Keywords:** Relation extraction - deep learning, Shortest dependency path

## Abstract

**Background:**

Extracting relations between important clinical entities is critical but very challenging for natural language processing (NLP) in the medical domain. Researchers have applied deep learning-based approaches to clinical relation extraction; but most of them consider sentence sequence only, without modeling syntactic structures. The aim of this study was to utilize a deep neural network to capture the syntactic features and further improve the performances of relation extraction in clinical notes.

**Methods:**

We propose a novel neural approach to model shortest dependency path (SDP) between target entities together with the sentence sequence for clinical relation extraction. Our neural network architecture consists of three modules: (1) sentence sequence representation module using bidirectional long short-term memory network (Bi-LSTM) to capture the features in the sentence sequence; (2) SDP representation module implementing the convolutional neural network (CNN) and Bi-LSTM network to capture the syntactic context for target entities using SDP information; and (3) classification module utilizing a fully-connected layer with Softmax function to classify the relation type between target entities.

**Results:**

Using the 2010 i2b2/VA relation extraction dataset, we compared our approach with other baseline methods. Our experimental results show that the proposed approach achieved significant improvements over comparable existing methods, demonstrating the effectiveness of utilizing syntactic structures in deep learning-based relation extraction. The F-measure of our method reaches 74.34% which is 2.5% higher than the method without using syntactic features.

**Conclusions:**

We propose a new neural network architecture by modeling SDP along with sentence sequence to extract multi-relations from clinical text. Our experimental results show that the proposed approach significantly improve the performances on clinical notes, demonstrating the effectiveness of syntactic structures in deep learning-based relation extraction.

## Background

Clinical texts such as discharge summaries and progress reports contain rich information of patients and are valuable data sources for many computerized clinical applications such as decision support systems. Although manual review can accurately transform unstructured narratives into structured data, it is costly and time-consuming, and thus, not feasible for applications that require extracting information from a large number of clinical documents. Therefore, natural language processing (NLP), which can automatically extract information of interest from narratives, becomes an enabling technology to support clinical researches and applications. One of the fundamental tasks of clinical NLP is to automatically extract relations between important clinical entities such as diseases, drugs, and lab tests. For example, in the sentence “likely **penicillin** and **sulfa** drugs leading to a **rash**”, recognizing that “rash” is an adverse event caused by the drugs “penicillin” and “sulfa” is very important to understand how the patient responded to the treatment.

Many approaches have been proposed for relation extraction tasks in the open domain [1], as well as for biomedical literature mining [2–5]. For clinical text, early systems primarily relied on rule-based approaches for relation extraction [6]. For example, Chen et al. [7] applied the MedLEE system [8] to extract relations between drugs and diseases, in order to facilitate building knowledge bases. Recently, with the development of annotated clinical corpora, increasing numbers of machine learning-based approaches have been developed for clinical relation extraction tasks [9–11]. Many of them have looked at identifying modifiers related to important clinical entities, e.g., signature of medications [12] and modifiers of diseases including negation, severity, temporal information etc. [13, 14]

An interesting relation extraction task was proposed in the 2010 i2b2/VA challenge, in which participating systems were asked to extract relations between important clinical entities (e.g., relations between diseases and drugs), rather than modifiers of these entities. Extracting such relations is critical for understanding patients’ disease, diagnosis and prognosis, as well as their treatments and outcomes. All the top-ranked systems used machine learning-based methods with extensive feature engineering. For example, Grouin et al. proposed a Support Vector Machine (SVM)based system with additional rules to capture linguistic patterns of relations [15]. Bruijn et al. investigated machine learning approaches with a focus on feature engineering, assessing large-dimensional features derived from both the text itself and other external sources [9]. They also performed a follow-up study by proposing a kernel-based model that consists of concept kernels, connection kernels, and tree kernels in order to capture lexical, semantic and syntactic features [16].

To avoid labor-intensive feature engineering and the high-dimensionality issue of features [17], deep learning-based architectures, which can automatically learn representations of data at multiple levels of abstraction, have been proposed and have demonstrated successes in multiple domains including medicine [18]. For the 2010 i2b2/VA relation extraction task, several deep learning-based approaches were also investigated. Sahu et al. [19] used convolutional neural networks (CNN) to learn features automatically. The model took a complete sentence with mentioned entities as input and each word in the sentence was represented with discrete features such as part of speech (POS) tag, chunk tag, etc. The system achieved an F-measure of 71.16% on a subset from the 2010 i2b2/VA challenge, which removed all the notes from the University of Pittsburg Medical Center and instances of 3 relation classes (TrWP, TrIP and TrNAP) from the whole dataset. Furthermore, Raj et al. [20] proposed a convolutional recurrent neural network (CRNN) model, which combines recurrent neural networks (RNNs) and CNNs to learn global and local context features. The model achieved a lower F-measure of 64.38% without using manual features. More recently, Luo et al. proposed the Seg-CNNs approach that splits the sentence into five parts: preceding, concept-1, middle, concept-2 and succeeding, and generates the representations for these five parts for relation classification, resulting in an F-measure of 74.2% on the original 2010 i2b2/VA challenge dataset [21].

Despite these related studies, deep learning-based methods for clinical relation extraction are still at their early stage of development and there is much room for improvement. One of the limitations of the current deep learning approaches for clinical relation extraction is that there is a lack of methods that can effectively represent and capture all the semantic and syntactic features from clinical sentences, especially long and complex sentences. In this study, we propose a new neural network architecture for clinical relation extraction, which integrates both sentence sequence and shortest dependency path (SDP) between the target entities into one deep learning framework. Our proposed model employs bidirectional long short-term memory network (Bi-LSTM) to capture semantic information from sentence sequence and uses CNN to generate local representations for all neighboring words in SDP. We evaluated this approach together with other baseline deep learning models using the 2010 i2b2/VA clinical relation extraction dataset and our proposed system achieved the state-of-the-art performance, indicating the effectiveness of this approach. To the best of our knowledge, this is the first study modeling SDP syntactic information together with sentence sequence in a deep learning framework for clinical relation extraction.

## Methods

### Dataset and preprocessing

We used a dataset from the 2010 i2b2/VA relation extraction challenge to develop and evaluate our models. The statistics of the dataset are shown in Table 1. The dataset contains 426 discharge summaries collected from 2 hospitals, with 8 relation types in total [10]. Please note that this dataset is a subset of the original dataset used in the challenge, since the University of Pittsburg Medical Center’s data were not available to the public and were removed from the original dataset after the challenge.

**Table 1:** – Statistics of the relation extraction dataset (a subset from the 2010 i2b2/VA challenge)

Relation type	Description	Number of instances
TeCP	Test conducted to investigate medical problem	504
TeRP	Test reveals medical problem	3052
PIP	Medical problem indicates medical problem	2203
TrCP	Treatment causes medical problem	526
TrAP	Treatment is administered for medical problem	2617
TrWP	Treatment worsens medical problem	133
TrNAP	Treatment is not administered because of medical problem	174
TrIP	Treatment improves medical problem	203
None	No relation between target entities	19,870
Total	–	29,282

As this is a relatively small corpus, individual words in clinical entities may have low frequency and may not have appropriate representation for training. Therefore, we replaced all entities with their entity types and used the updated sentences for training. We also added “tar-” and “ent-” to denote target entities and non-target entities in the sentence, respectively. For example, the instance “She was maintained on [an epidural]treatment and [pca]treatment for [pain control]problem” was converted to “She was maintained on tar_treatment and ent_treatment for tar_problem”, where “tar_treatment” and “tar_problem” are the target entities and “ent_treatment” is a non-target entity that we did not take into consideration in this instance. The replacement also introduced the semantic information about entity types into the model.

### Our approach

**Fig. 1:**
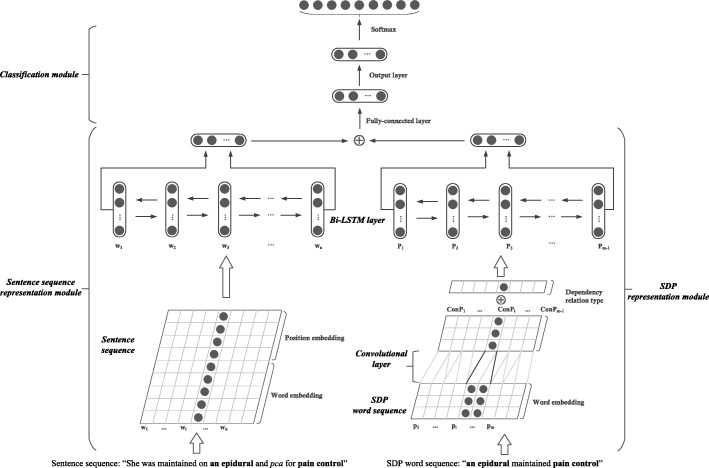
- Architecture of our model. Our neural network architecture consists of three modules: (1) sentence sequence representation module; (2) SDP representation module; and (3) classification module

As shown in Fig. 1, our neural network architecture consists of three modules: (1) sentence sequence representation module, which takes the entire sentence along with position features as the input and generates the representation of the sentence by using a Bi-LSTM network; (2) SDP representation module, which implements the CNN and Bi-LSTM network to capture the syntactic context for target entities using SDP information; and (3) classification module, which concatenates outputs of both of the previous modules into a context vector with a fully-connected layer and feeds it into the output layer with the Softmax function for classification.

### Sentence sequence representation module

We used S = {w1, w2 …, wn} to denote the word sequence of a sentence. Each word wi is represented by both word embedding and position embedding. Word embedding maps words into a low-dimensional space to capture semantic information among words [17] . It has been widely used as the input of the neural networks in NLP tasks. In this study, we employed the word2vec [22] to pre-train word embeddings using the Medical Information Mart for Intensive Care (MIMIC)-III clinical corpus [23]. Besides the words, the positions of the target entities in the sentence also play an important role in relation extraction. Therefore, we used position embeddings to represent the position information of target entities, which is adapted from Zeng et al. [14]. For example, in the sentence “She was maintained on [an epidural]treatment and [pca]treatment for [pain control]problem”, the relative distances of “She” to “[an epidural]treatment” and “[pain control]problem” are − 4 and − 8, respectively. In our model, we mapped the relative distances to vectors and initialized them randomly. The sentence representation was further fed to the Bi-LSTM network, which consists of a forward LSTM and a backward one. The output ${h}_f^{(t)}$ and ${h}_b^{(t)}$ of the forward and backward LSTMs were then concatenated into ${h}^{(t)}=\left[\ {h}_f^{(t)},{h}_b^{(t)}\right]$ which is the output vector of Bi-LSTM.

### SDP representation module

**Fig. 2:**
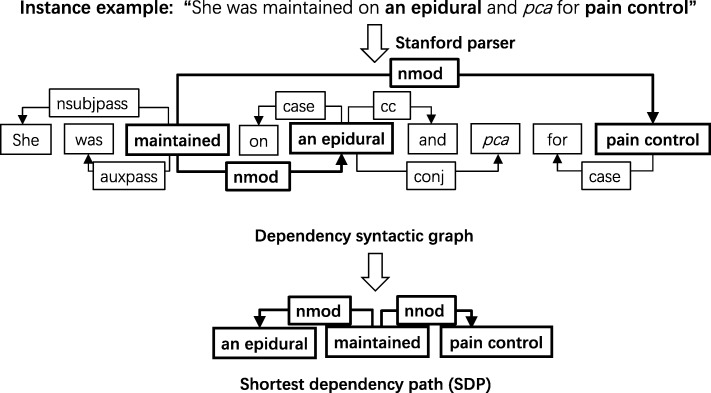
- An illustration of SDP generation. This figure shows the dependency syntactic graph and the SDP of sentence “She was maintained on a epidural and pca for pain control”

Several recent studies have shown that the SDP can boost the performance of the relation extraction [24–27]. In clinical relation extraction, we also observed that the SDP between entities provides strong hints for determining the relationship. For example, in Fig. 2 the dependency syntactic structure of a sentence can be represented as a graph and there is always a shortest path between two words in the graph. The SDP between the target entities ‘an epidural’ and ‘pain control’ is:

[**an epidural**]_treatment_ – nmod → maintained–– nmod → [**pain control**]_problem_.

The words “maintained” in the SDP provide critical information for classifying the relations between the target entities of “an epidural” and “pain control”. Besides the words on the path themselves, the type of dependency relation between the two neighboring words is also useful. In the example, the dependency relation ‘nmod’ indicates that the word “pain control” is the noun compound modifier of the word ‘maintained’, which provides supplemental information for relation classification. Thus, the relation extraction benefits from the semantic information contained in the representation of the words in SDP, as well as syntactic information in SDP, especially for the long and complex sentences. In this study, we used the Stanford parser to parse the sentences and generate dependencies.

Following the above intuition, we also designed a neural network to model SDP. We used P = {p1, p2, …, pm} to denote the word sequence of the SDP. Each word pi in the SDP is represented by its word embedding. We utilized the convolutional approach [28, 29] as expressed by Eq. (1) to merge the two neighboring words that contain a certain dependency relation:

1 $$ {ConP}_i=\left[\begin{array}{c}{P}_i\\ {}{P}_{i+1}\end{array}\right]\bullet \mathrm{M} $$

where *P*_*i*_ is the embedding of word pi (*i* = 1, 2, …, m), and ${M}^{n_1\times {n}_2}$ is the transformation matrix that is the same across all local features in the SDP. ${ConP}_i\in {R}^{2\times {n}_2}$ is the transforming result of the two neighboring words using M, where *n*_1_ is the dimension of word embeddings and *n*_2_ is a hyper-parameter that denotes the output dimension after convolutional transformation. After the transformation, the representation of SDP is ConP = {*ConP*_1_, *ConP*_2_, … , *ConP*_*m* − 1_}. We used {d1, d2, …, dm-1} to denote the dependency relation types between all neighboring words and each dependency relation type was randomly initialized into a vector. The output of the convolutional layer and the embeddings of dependency relation types were concatenated and fed to a Bi-LSTM network to generate the SDP representations.

### Classification module

In the classification module, we first concatenated the outputs of the sentence sequence representation module and the SDP representation module, and then fed it to a fully-connected layer to generate the context vector. Finally, the context vector was fed to an output layer with the Softmax function to classify the relation between the candidate entities. The probability of a candidate pair belonging to a relation type was calculated as follow:

2 $$ \mathrm{p}\left(\mathrm{i}|\mathrm{s}\right)= softmax\left({W}_o\bullet s+{b}_o\right) $$

where *W*_*o*_ and *b*_*o*_ are the weight parameters, and s is the feature representation of the candidate pair. In our method, we used the cross-entropy cost function as the training objective function. Adaptive moment estimation (Adam) [30] was used to optimize the parameters in our model with respect to the objective function.

### Experiments and evaluation

We performed a 5-fold cross-validation using the dataset from the challenge and reported micro-average precision, recall, and F-measure from the 5-fold cross validation results. In our experiments, we used the Pytorch library [31] to implement our proposed model. The dimensionality of the word embeddings and position embeddings were set to 100 and 50, respectively. The hidden unit number of Bi-LSTMs and the SDP-based convolutional layer was 200. The learning rate of Adam was 0.00001 and the mini-batch size was set to 32. To alleviate overfitting of the model, we also used dropout [32] to randomly drop units and their connections from the fully-connected layer in the model during training.

## Results

As shown in Table 2, our method achieved an F-measure of 71.84% when only the sentence sequence module (with both word embedding and position embedding) was used. When we added the SDP representation module (both word sequences and relation type), the system achieved the best F-measure of 74.34%, with an increase of 2.50%. Our results also showed that both word sequences and the dependency relation types of SDP contributed to the increase of performance.

**Table 2:** – Performance of our proposed methods on the 2010 i2b2/VA subset (5-fold cross validation)

Features	Precision (%)	Recall (%)	F-measure (%)	∆ (%)
Sentence Sequence only	74.01	69.79	71.84	–
+SDP (Word Sequence)	74.20	72.84	73.51	1.67
+SDP (Word Sequence + Relation Type)	75.69	73.03	74.34	2.50

To further evaluate the effectiveness of SDP features, we looked at the F-measures achieved before and after adding SDP features for each relation type in Table 3. It is clear that SDP features improved performance for every type of relations. For some relation types such as TrWP, TrNAP and TrIP, the improvements were dramatic (e.g., 26.52% increase for TrWP).

**Table 3:** – Improvements in F-measure by adding SDP module for each relation type

Relation Type	Sentence Sequence	Sentence sequence + SDP	∆
TeCP	54.24	61.17	6.93
TeRP	83.64	84.44	0.80
PIP	63.09	63.33	0.24
TrCP	56.45	62.13	5.68
TrAP	75.53	79.74	4.21
TrWP	18.05	44.57	26.52
TrNAP	30.49	42.27	11.78
TrIP	51.85	61.59	9.74

## Discussion

In this study, we propose a novel neural network architecture to model syntactic structures (SDP) along with sentence sequences for clinical relation extraction. Experimental results show that our proposed method outperformed the baseline method that used sentence sequence only, demonstrating the value of incorporating SDP features into deep learning-based approaches for clinical relation extraction. In Table 4, we compare our approach with the previously published systems in terms of performance on the same 2010 i2b2/VA challenge dataset. The first five studies used exactly the same dataset as ours and our approach apparently achieved a much higher performance than those reported previously. The last study by Luo et al. [21]was published recently, in which they achieved an F-measure of 74.2% by using the original dataset from the challenge (871 notes in total). Although our dataset is much smaller than what they used (426 vs. 871 notes), our approach actually achieved slightly better performance as theirs (F-measure 74.34% vs. 74.2%respectively).

**Table 4:** – Comparison of performance of different systems reported on the same i2b2–2010 corpus

Publications	Models	Precision (%)	Recall (%)	F-measure (%)
Rink et al. [33]	SVM	67.44	57.85	59.31
Sahu te al. [19]	Multi-CNN-Max	55.73	50.08	49.42
Sahu and Anand [34]	LSTM-ATT	65.23	56.77	60.04
Wang et al. [35]	RCNN	50.07	45.34	46.47
Raj et al. [20]	CRNN	67.91	61.98	64.38
*Luo et al. [21]	Seg-CNN	–	–	74.20
	Our model	75.69	73.03	74.34

*Luo et al. used the original dataset from the challenge (871 documents in total)

We also conducted an analysis to further illustrate why SDP could help clinical relation extraction. Table 5 shows several examples that were classified into wrong relations when only sentence sequences were used. After integrating the SDP features, these relations were correctly recognized. We summarize possible reasons that lead to the success of the proposed model as follows: 1) The length of SDP is much shorter than the length of the whole sentence sequence, which may reduce noise caused by many other entities; 2) SDP emphasizes more on syntactic structures, which are critical to the relation extraction task; and 3) The dependency relation type represents valuable syntactic relation information between the two neighboring words in the SDP.

**Table 5:** – Instances Corrected by Adding SDP-based Module

Relation Type	Sentence Sequence	SDP
TrWP	Subsequent discontinuance of [*azithromycin*]_treatment_, *[trial_of_5-fc]*_treatment_, with *[increasing neutropenia]*_problem_ requiring discontinuance, change if [*itraconazole*]_treatment_ to [*voriconazole*]_treatment_, given [*continued neutropenia*]_treatment_, and trial of [*sulfadiazine*]_treatment_, discontinued for [*increasing ars*]_treatment_	*[trial_of_5-fc]*_treatment_ – appos →[*azithromycin*]_treatment_ – nmod →discontinuance– nmod →*[increasing neutropenia]*_problem_
TrNAP	*[His cast]*_treatment_ was removed by the orthopedic service in anticipation of [*this edema*]_problem_ and to avoid *[compartment syndrome]*_problem_	*[His cast]*_treatment_ –nsubjpass→ removed – nmod →service– acl → avoid –dobj→ *[compartment syndrome]*_problem_
TrIP	[*His hypertension*]_problem_; *[his high blood pressure]*_problem_ was controlled with *[intravenous nitroglycerine]*_treatment_ in the early going and then he was switched to [*an oral regimen*]_treatment_ for better control after he was removed from the intensive care unit	*[his high blood pressure]*_problem_ –nsubjpass→controlled– nmod →*[intravenous nitroglycerine]*_treatment_

The italics in each sentence sequence are the candidate pair entities

Although our method achieved the state-of-the-art performance on the 2010 i2b2/VA dataset, we believe that there are many ways to further improve deep learning-based relation extraction in clinical text and we plan to investigate the other aspects in our future work. One of the directions is to leverage existing knowledge bases to improve the accuracy of deep learning models. For example, we plan to study distant supervision methods under the context of deep learning architectures for relation extraction in the medical domain. By automatically generating training data via aligning knowledge bases and texts, we can assume two entities that have a relation in the knowledge bases will express the same relation in a sentence. The knowledge of clinical relations can be used to automatically annotate the dataset, in order to reduce the cost of manual curation [36, 37].

## Conclusion

In this study, we propose a new neural network architecture to extract multi-relations from clinical text by modeling SDP along with sentence sequence. Our experimental results show that the proposed approach achieved significant improvements over comparable existing methods on the 2010 i2b2/VA relation extraction task, demonstrating the effectiveness of syntactic structures in deep learning-based relation extraction.

## Abbreviations

Adam: Adaptive moment estimation
Bi-LSTM: Bidirectional long short-term memory
CNN: Convolutional neural network
CRNN: Convolutional recurrent neural network
MIMIC: Medical Information Mart for Intensive Care
NLP: Natural language processing
POS: Part of speech
RNN: Recurrent neural network
SDP: Shortest dependency path
